# A Trial of Student Self-Sponsored Peer-to-Peer Lending Based on Credit Evaluation Using Big Data Analysis

**DOI:** 10.1155/2019/9898251

**Published:** 2019-04-16

**Authors:** Yujiao Hou, Xiaofeng Ma, Guang Mei, Ning Wang, Weisheng Xu

**Affiliations:** College of Electronics and Information Engineering, Tongji University Shanghai, Shanghai, China

## Abstract

There is still no effective approach to overcome the problem of credit evaluation for Chinese students. In absence of a reliable credit evaluation system for students, the university students have to only apply through online peer-to-peer (P2P) loan platforms because Chinese financial institutions typically reject students' loan applications. Lack of students' financial records hinders financial institutes and banks to routinely evaluate the students' credit status and assign loans to them. Hence, this paper attempted to benefit from university students' diversified daily behavior data, and logistic regression (LR) and gradient boosting decision tree (GBDT) algorithms were also used to develop robust credit evaluation models for university students, in which the validation of the proposed models was assessed by a real-time P2P lending platform. In this study, the students' overdue behavior in returning books to university library was used as an index. With training 17838 samples, the proposed models performed well, while GBDT-based model outperformed in identification of “bad borrowers.” Based on the proposed models, a self-sponsored peer-to-peer loan platform was established and developed in a Chinese university for ten months, and the achieved findings demonstrated that adopting such credit evaluation models can effectively reduce the default ratio.

## 1. Introduction

The higher education system in China has been rapidly developed since 1990s. In 2017, the number of Chinese students who have enrolled in higher education system has reached 46.10 million. There is a broad business development space in the student credit market in the global scale. Unlike students in Western countries, Chinese students are typically forbidden to reach credit cards through Chinese banks until they are graduated and could be able to find a proper job [1]. Besides, running business credit development programs for young individuals is indeed slow due to their poor credit records. According to the broad business development space, several Chinese microlenders have legally launched loan services to university students [2]. Due to the lack of students' credit information, Chinese companies do not properly manage the associated risks. Hence, these companies usually provide high-interest loans which are economically unbeneficial to university students [3]. High interest rates have caused lots of negative consequences, and students' debts continue to pile up and lots of students fell into debt crisis [4]. To get rid of heavy debts, numerous students were forced to prostitute, and even some of them have suicided [5]. Therefore, it is of great importance to develop an effective and robust credit evaluation system for university students and then present that system to financial institutions.

It is well known that data mining is the process of discovering patterns in large datasets. Chinese students typically live in universities' dormitories, which is less common among students in Western countries. A Chinese student can easily purchase different items, access the Internet, and eat in various canteens using his/her campus card. The campus card can also be an entry card to enter sports clubs, library, and dormitory as well. Swiping cards produce plenty of records, including consumption record, in- and out-record, etc. These patterns and consumption data are typically collected and stored by student administration offices of a university. In this paper, it was attempted to utilize such data to develop a credit evaluation system. These datasets vary in formats and are remarkably different from financial transactions, which are routinely used by classical credit evaluation methods.

This paper develops a credit evaluation model for Chinese students using the multisource dataset provided by an anonymous Chinese university, and the developed method is validated by a real-time peer-to-peer (P2P) lending platform.

## 2. Background of Credit Evaluation Methods and P2P Lending Service Providers

In recent years, data mining techniques have been used in credit scoring systems. Dave Girouard, the CEO of Upstart company, believes that there are loopholes in the current credit system. Upstart is an American P2P credit agency launched in May, 2014, and has facilitated on approving 8700 loans, totally $12.50 million in 2014. This company believes that traditional credit score systems cannot properly describe young clients' repayment condition, thus they employed a robust big data method to assess students' level of education, background, and work experience [6]. ZestFinance, an American company presenting end-to-end technology platform and underwriting expertise to financial firms around the world, mainly focuses on analyzing and estimating customers' potential especially those people with poor credit records [7]. In China, Alibaba Group Holding Limited has built a fraud risk monitoring and management system based on real-time big data processing technique and an intelligent risk model [8]. Compared with traditional credit assessment methods (e.g., financial information derived from bank loans, credit cards, mortgages, and hire-purchase) [9, 10], big data methods not only rely on the history of financial information but also investigate diverse data such as social communication, service performance, and behavioral characteristics on the Internet [11]. By analyzing these heterogeneous data, individual's credit can be inferred based on the level of customer's essence, e.g., personal character, psychology, and morality, which is more significant than judgement on the basis of financial records, and can assist those people who may suffer from low credibility [12]. Different credit scoring techniques have been used to establish credit models. The most commonly used algorithm for credit evaluation in banking system is logistic regression [13, 14]. Besides, decision tree is a well-known algorithm to predict individuals' credit, such as credit card fraud [15]. In spite of remarkable accuracy and simple construction of the algorithm, a credit model based on logistic regression method possesses strong interpretability, which is indeed favorable in banking system [16]. While new techniques have shown superior accuracy for credit prediction, they have not been widely used in practice yet. For instance, artificial neural network (ANN) has been found to be superior than logistic regression in terms of accuracy [17, 18]; however, it is typically criticized because of its poor performance when processing irrelevant or small datasets. These methods, e.g., support vector machines (SVMs) and neural networks, may lead to a better classification performance; however, they still suffer from some poor characteristics making them more vulnerable and unreliable [19]. Moreover, these two methods are always described as black box because they do not present any information about functional relationship with features, which is an important disadvantage for banking system to reject clients' loan applications without any reasonable reasons [20]. Simultaneously, some linear classifiers, such as linear discriminant analysis (LDA) and multilayer perceptron (MLP), a model of neural networks, have reported satisfactory results [21].

There are several hybrid systems that combine conventional algorithms together to improve classification ability. For example, to estimate the influence of the state of economy on loan losses, a linear regression method was combined with SVM, and this two-stage hybrid approach outperformed other techniques on prediction [22]. A two-stage hybrid method based on ANN and multivariate adaptive regression splines (MARS) was presented in [23]. After using MARS in developing a credit scoring model, the obtained variables were then served as inputs for the ANN. However, significant improvements were not observed. Particle swarm optimization (PSO) was used to optimize the parameters required for SVM in credit scoring; compared with backpropagation (BP) neural network method, the hybrid PSO-SVM algorithm possesses a higher accuracy and remarkably lower type of the error which can avoid huge financial cost [24]. Ensemble systems have been developed to perform better than usual models on the same datasets. Two conventional ensemble learning methods are bagging and boosting, utilizing an ensemble of weak classifiers to create a strong classifier. Among all boosting methods, AdaBoost is a machine learning meta-algorithm, and its performance is on the basis of repeating rounds of boosting iterations [25]. For each iteration, the dataset is sampled based on the calculated weights, and a proper weak classifier is optimally found dividing the sampled data into the classes. The weight is then assigned to the selected weak classifier based on the mechanism of the data division. The combination of AdaBoost with BP neural network has outperformed than a single-layer neural network and a traditional AdaBoost algorithm [26]. Based on AdaBoost algorithm, Friedman developed a general gradient descent boosting paradigm for additive expansions based on any fitting criterion, which could reduce residual error by establishing new models on the gradient direction in each iteration. Gradient boosting machine is widely used in regression and classification problems, possessing an outstanding performance [27]. Gradient boosting machine is widely used in regression and classification problems, which possessed an outstanding performance [28]. In the present study, it was attempted to adopt both logistic regression (LR) and gradient boosted decision tree (GBDT) to develop credit evaluation models.

Additionally, to experimentally validate the developed credit model, the authors have implemented a P2P lending platform offering loans to students. Architecture of P2P reflects that users can directly connect with each other in order to share their information or lending-based issues [29]. In such social networks, peer effect tends to be more influential on people's behavior [30]. Several high-profile papers examining obesity pointed out the importance of obesity's influence (or “contagion”) that travels along social networks [31–33]. Also, lifestyle can also be socially contagious. Sinan Aral and Christos Nicolaides found that exercise was contagious in social networks, and its contagiousness varied with the relative activity of and gender relationships between friends [34]. Escardíbul et al. found that peer consumption had a positive effect on youth's console and Internet use [35]. Dean Eckles et al. [36] used a peer encouragement design to estimate the effects of receiving feedbacks from peers on posts shared by focal individuals in Facebook, and they found that receiving additional feedback causes individuals to give feedback to others and to share new posts. For students, it has been proved that peers and social interaction had great effect on academic performance [37, 38]. In addition, in group lending, Li et al. [39] found that the likelihood of a member making a full repayment would be 15 percent higher on average if all the other fellow members made full repayment compared to the case where none of the other members repaid in full. In recent years, peer-to-peer lending networks have been popular among small- and microenterprises [40], and the transactions are processed through the Internet excluding the involvement of collateral by financial institutions [41, 42]. For P2P lending, the information of both loan and debt/income ratio of a borrower will affect the final interest rate of a loan [43]. Besides, numerous scholars reported that the social relationships of a borrower can affect loan success, interest rate, and defaultable debt [44].

The rest of the paper is organized as follows.  Section 3 describes data processing and feature extraction, including the definition of “bad borrowers.” Section 4 explains the credit evaluation process, involving the evaluation criteria and development of a credit model, and compares two credit models' performance. The P2P lending platform is detailed in Section 5, including the basic rules and operation of the P2P lending platform, as well as the findings achieved.

## 3. Data Processing and Feature Extraction

This research is based on a dataset provided by an anonymous Chinese university; after feature extraction, the data are transformed and loaded into the data warehouse. The dataset includes 78716461 items of 31586 students who have enrolled during the years of 2013 and 2015 in two campuses. Besides, it involves several aspects: basic information, library loan records, records of entrance to the library and dormitory, grades, consumption records, and scholarship records. All data were kept confidential to protect students' privacy, and the students were empowered with their own online data.

Real-time datasets are susceptible to various quality issues, such as missing values, different data structures, data redundancy, and imbalanced data [45]. Herein, standard preprocessing operations are applied to the data. In this study, after a comprehensive review of raw data, both mean imputation and case deletion were adopted to deal with missing values, and all outliers were removed as well. In order to shun subjectivity, one-sidedness, and superficiality in model progress, there was no assumption before mining data because it was not feasible to accurately determine which factor would affect the dependent features in advance. Hence, it was attempted to design several features. Eventually, 29 features of four perspectives were designed, including students' personal information, library borrowing information, daily life data, and transaction records. Before applying these features to subsequent analyses, it should be attempted to standardize all features. The features are accordingly standardized using *Z*-score method, and the *Z* vectors can be obtained using the following equation:

(1) $$\begin{array}{c} Z_{i}=\frac{X_{i}-\bar{X}}{S_{i}}, \end{array}$$

where $\bar{X}$ is the mean value and *S*_*i*_ denotes the standard deviation of the *i*_th_ feature. The detailed information of all features after data preprocessing is listed in Table 1.

In different application fields, the definition of a “bad customer” accordingly varies. Generally speaking, for a credit risk management, a “bad customer” typically implies a customer with high possibility of default [46]. In banking system, several credit rating models have been developed in order to classify customers into committed or uncommitted. If the rate of accuracy of classification increases, banks and financial institutions can optimally implement a merit-based loan assign system to different applicants.

In this paper, the university students were assessed in absence of their financial credit information. Regarding all behaviors happened in the university, behavioral data were collected by university's information center, and data were then processed by the system. It was revealed that there are numerous similarities between borrowing a book from library of university with loan borrowing or utilizing a credit card. To scrutinize the process of borrowing a book from a library, it is important to note that a book must be returned by the due time or the due date, and an overdue book will incur fines. In the university, a student is permitted to borrow only 10 books for 30 days, and if a student cannot return those books on time, he/she will be fined 0.5 CNY per each day and each book. To prevent overdue, a student can apply online only for one time to postpone the deadline for 15 days, while the request can be only submitted once per each book. Thus, if a student could successfully register his/her deferral request online, he/she will be allowed to take out a book for 45 days without any penalty. When the penalty reaches 5 CNY, a student will be prohibited to borrow books for 3 months. Similar to a bank or financial institution that regularly sends reminder messages to a debtor who has received a loan, the library of a university also has a reminding system to inform the students to return the books on time through automatically forwarding daily e-mails since 7 days before the deadline. Therefore, a “bad borrower” here is defined as an individual who has the highest frequency of overdue in terms of returning books which were borrowed from a library.

Herein, the example on returning books to a library is taken as a symbol of borrowing behavior into consideration, and an index of “bad borrower” is defined that is widely used in credit evaluation in banking system. There is a common method in banking system to decide whether a customer's loan/credit card application is passed or rejected. For this purpose, first of all, all customers' credibility should be ranked by the forecasted probability of default which is given based on banks' credit models. Then, top 5% of customers with the highest default probability are considered as “bad customers,” and their loan/credit card applications are accordingly rejected. Similarly, in this study, it was attempted to pick out students who had default records of returning books, and they were divided into twenty groups and were ranked by a variable named *Lib_borrow_exceed_cnt* (denoting the frequency of returning overdue books), in which each rank involved the same number of students. A variable called *label* was also defined to indicate whether a student is a “good borrower,” who is denoted by 0 or a “bad borrower,” who is denoted by “1.” In this study, it was attempted to consider the students in the top group (with the highest frequency of returning overdue books) as “bad borrowers” (who are denoted by “1”), and the remaining include 0-default students as “good borrowers.” Students with no record of borrowing books were removed from dataset.

Before modeling, it was attempted to refine features in order to eliminate their dependencies. Herein, Pearson's correlation coefficient was used to refine features. After refinement, 13 features were selected as follows: *Prob_Back*, *Prob_Nation*, *Lib_borrow_total_cnt*, *avg_grade*, *Prob_Sex*, *excellent_rate*, *Lib_borrow_avg_exceed_cnt*, *Lib_renew_prob*, *Lib_borrow_avg_exceed_time*, *fail_rate*, *avg_cost*, *sum_transaction_amount*, *mean_recharge*. These features are explained in Table 1.

To develop credit evaluation models, all the processed data were categorized into “training set” and “test set.” After preprocessing, 29741 students remained, including 487 “bad borrowers” and 29254 “good borrowers.” In addition, 288 “bad borrowers” and 17550 “good borrowers” were randomly assigned to training set, and the test set involved 240 “bad borrowers” and 14625 “good borrowers.” Eventually, 41 “bad borrowers” and 2434 “good borrowers” were assigned to both training set and test set. Owing to the skewness of dataset, it may cause overfitting of models. Thus, for both sets, it was attempted to randomly pick out 200 “bad borrowers” as well as 3800 “good borrowers” from their datasets at every turn, and the process was iterated for 20 times, until 20 training groups and 20 test groups would be reached. Here, after developing credit evaluation models, the students were generally divided into “bad borrowers” vs. “good borrowers” based on each individual's default probability, and the outputs showed that the proportion can be eventually reached at 5 : 195. It could effectively reduce the overfitting on nondefault set and cover the entire dataset as far as possible by means of the mentioned method.

## 4. Credit Evaluation Model Based on Big Data Analysis

### 4.1. Criteria for Credit Evaluation Model

It is necessary to investigate the efficiency of the credit evaluation model. Percentage of correctly classified instances (including accuracy (ACC), true positive (TP) rate, true negative (TN) rate, false positive (FP) rate, and false negative (FN) rate) and receiver operating characteristic (ROC) curve are two conventional evaluation metrics. Accuracy calculates the ratio of correctly classified cases to the total number of cases [47], which is undoubtedly the most frequently used metric. In this research, ACC is defined as the percentage of correctly predicted borrowers among all borrowers. The ACC is given by

(2) $$\begin{array}{c} \text{accuracy}=\frac{\text{TP}+\text{TN}}{\text{TP}+\text{FN}+\text{TN}+\text{FP}}, \end{array}$$

where sum of TP and FN is equal to the total number of correct predictions, whereas sum of TP, FN, FP, and TN is equal to the total number of predictions.

Besides ACC, the sensitivity (or the true positive rate) which measures the proportion of actual positives that are correctly detected and specificity which measures the proportion of actual negatives that are correctly detected are also studied. In this study, sensitivity is defined as the percentage of correctly predicted “bad borrowers” among true “bad borrowers.” The sensitivity is expressed as

(3) $$\begin{array}{c} \text{sensitivity}=\frac{\text{TP}}{\text{TP}+\text{FN}}. \end{array}$$

The specificity (or the true negative rate) is defined as the percentage of correctly predicted “good borrowers” among true “good borrowers.” The specificity is given by

(4) $$\begin{array}{c} \text{specificity}=\frac{\text{TN}}{\text{FP}+\text{TN}}. \end{array}$$

As an example, we examine a demo to show the necessities to adopt these two criteria. Consider the following two misclassification tables as shown in Tables 2 and 3. The ACC of two results are both 94.5%. However, in Table 2, there are 45 out of 50 “bad borrowers” successfully detected, while in Table 3, only 25 out of 50 “bad borrowers” are detected. The sensitivity of the first example is 90% while the second example's sensitivity is only 50%. In the financial industry, the loss of misclassifying “bad customers” is generally regarded as 5–20 times higher than the loss caused by misclassifying “good customers” [48]. Although the specificity of the second example (96.8%) is slightly higher than that of the first example (94.7%), the actual loss of the second example is still much higher than that of the first one. Hence, only ACC is not enough to evaluate models without sensitivity and specificity.

In addition, the ROC curve which was proposed by Hanley and McNeal in 1982 was herein adopted as well. The ROC curve is created by plotting the TP rate versus the FP rate at various threshold settings [49]. The TP rate is known as sensitivity, and the FP rate is also defined as the fall-out or probability of false alarm and can be calculated as (1 − *specificity*) [50]. Area under the ROC curve (AUC) was used to evaluate the performance of a binary classification system [51]. In this study, ACC, sensitivity, specificity, and ROC were used to measure the prediction performance of the developed models.

### 4.2. Modeling

#### 4.2.1. Logistic Regression

Logistic regression is one of the most widely used techniques in statistical analysis, and it has possessed fewer classification errors in credit risk assessment [52]. Assume that we have a training set *T*, including *n* students, e.g., *X*=(*X*_1_, *X*_2_,…, *X*_*n*_), and each student has a feature vector involving *j* descriptions, e.g., *X*_*k*_=(*X*_1*k*_, *X*_2*k*_,…, *X*_*jk*_), (*k*=1,2,…, *n*), *x*_*k*_ ∈ *R*. Thus, the particular form of logistic regression model is

(5) $$\begin{array}{c} \pi \left(x\right)=P\left(Y=\frac{1}{x}\right)=\frac{\exp\left(wx\right)}{1+\exp\left(wx\right)}. \end{array}$$

The transformation of the *π*(*x*) logistic function is known as logit transformation:

(6) $$\begin{array}{c} g\left(x\right)=\ln\left[\frac{\pi \left(x\right)}{1-\pi \left(x\right)}\right]=wX,\\ w={\left(w^{\left(1\right)},w^{\left(2\right)},\ldots ,w^{\left(n\right)},b\right)}^{T},\\ w^{\left(1\right)}=\left(\beta_{0},\beta_{1},\ldots ,\beta_{n}\right),\\ x={\left(x^{\left(1\right)},x^{\left(2\right)},\ldots ,x^{\left(n\right)},1\right)}^{T}. \end{array}$$

Then, those 13 features were forwarded into LR model, and the stepwise regression method was used to do further refinement. After that, 5 features that were not found significant were eliminated. The maximum likelihood estimates of the features remained can be found in Table 4.

After training by the training set (involving 288 “bad borrowers” and 17550 “good borrowers”), the LR model can be formulated as

(7) $$\begin{array}{c} a=\ln\frac{p}{1-p},\\ p=\frac{\exp\left(a\right)}{1+\exp\left(a\right)}, \end{array}$$

where *p* denotes the probability of being a “bad borrower” ranging from 0 to 1.

Afterwards, all the students were ranked by *p*, and the top 5% of students were predicted as “bad borrowers,” while others were predicted as “good borrowers.”

#### 4.2.2. Gradient Boosting Decision Tree

As an ensemble learning method, GBDT is a successful algorithm and is applied to classify the numerical problems, that constructs a composite classifier by sequentially training differentiated classifiers in the gradient direction for reduction of the residual error, while that remarkably emphasizes on certain patterns [53]. For the credit scoring problem, a training set is given *S*={*x*_*k*_, *y*_*k*_}_*k*=1_^*N*^, where *x*_*k*_ ∈ *R*^*m*^ is input data and corresponding output is denoted by *y*_*k*_. The main goal is to find out an approximation $\hat{F}\left(x\right)$ of function *F*(*x*) that could minimize the expected value of a particular loss function *L*(*y*, *F*(*x*)) [54]. Initially, an equal weight is assigned to each sample in *S*, meaning that each sample has the same selection opportunity at the first step. Generating *T*-decision tree classifiers for the model requires *T* rounds of trained decision tree with *T* different training sample groups *S*_*t*_(*t*=1,2,…, *T*). In round *t*, the function to determine the weight of sample *k* is denoted by *D*_*r*_(*k*). In each round, after the construction of classifier *M*_*t*_ which provides a function *F*_*t*_ to map *x* to {−1, 1}, the value of *D*_*r*_(*k*) is adjusted in terms of classification pattern by classifier *M*_*t*_, and the training sample group *S*_*t*+1_ is then generated in terms of *D*_*t*_ on *S* with sample replacement.

### 4.3. Prediction Performance of the Developed Algorithm

Here, the prediction performance of the two models is discussed. In the present study, a test dataset was used comprising of 14864 samples (with 14625 “good borrowers” and 240 “bad ones”). As mentioned above, to elude the skewness of data, 20 test groups were extracted, in which each group contained 200 “bad borrowers” and 3800 “good borrowers.” Tables 5 and 6 list the prediction results of the LR- and GBDT-based models on the 20 test groups, respectively. Apparently, both models performed excellent on the prediction, and the ACC of two models is almost equal. On the problem of identification of “bad borrowers,” the sensitivity of GBDT-based model is slightly higher (about 7%) than LR-based model, which means the GBDT-based model has a slightly better ability on detecting “bad borrowers.”

**Figure 1:**
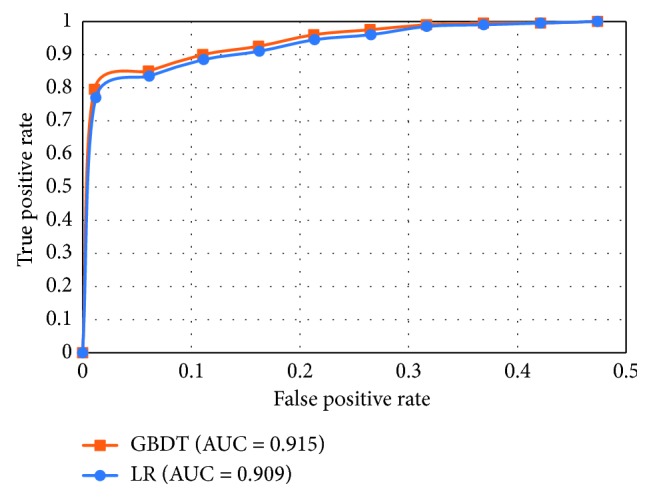
The ROC curves for the LR model and the GBDT model.

To further assess continuous predictive performance of these two models, ROC curves were plotted. We selected one test group and ranked all the samples in 20 groups in descending order (each group contained 200 samples) by the default rate generated by LR-based model. After that, the cumulative ratio of true “bad borrowers” and “good borrowers” was calculated. The same process was undertaken for GBDT-based model on the same test group. Tables 7 and 8 show the prediction performance of LR- and GBDT-based models. According to the result of Tables 7 and 8, the ROC curves of the two models were drawn. Based on Figure 1 and the value of AUC, it can be seen that the AUC of GBDT-based model is only 0.006 greater than LR-based model, which means the difference of two models' ability on detecting “bad borrowers” is negligible. In general, the LR-based model and the GBDT-based model are both efficient models, while GBDT-based model outperforms a little; the difference is still very small so that the two can be used interchangeably.

## 5. P2P Lending Experiment Based on the Credit Evaluation Model

So far, we cannot confirm that the default records of borrowing books can denote poor personal credit especially on financial issues. If it did not make sense, the credit evaluation models we have built would be meaningless. Therefore, a P2P lending platform was developed based on our proposed credit evaluation model. The platform was implemented within the campus LAN (local area network) and can only be accessed by university students.

### 5.1. Fundamental Rules of the P2P Lending Platform

To get a loan from the platform, a student must register with his/her student ID and phone number and agree with our terms of service. Behind the scenes, every registered user's credit score was calculated automatically. Considering that the nature of this loan service was for experiments, loans provided by the platform were all free of interest. The period of a loan was 30 days, and the user would get a bonus point if he/she repaid from the 14th day to the 30th day. The bonus point was designed to prevent those who did not have actual demanding for loan, showing up as lending and repaying in one single day or a quite short period. There was no explanation about the function of bonus point, but it turned out effective. There were only 31 out of 258 payments occurred from the first day to the 13th day. As soon as a user repaid on time (in 30 days), there would be no interest or service fee; otherwise, he/she would have to pay service fee which was 1% of the principal. There was a reminder system which can automatically send messages to remind debtors to repay, and it worked in the last 3 days of every loan period. In addition, if a student did not repay on time, we would give him/her a call from the 33rd day after his/her loan day until the day he/she repaid. The funds of this platform were raised from anonymous students in the same university.

We adopted LR-based model's results to calculate credit scores. Although both credit evaluation models had strong capability and were totally interchangeable, the reason why we chose LR-based model instead of GBDT was that the parsimony and computation speed of LR model are better. The credit score was equal to (50  *∗*  (1 − *p*)+50), where *p* was the output of LR model which meant the probability of default. According to the rules of credit model, users whose credit scores were the lowest 5% should be regarded as “bad borrowers,” which meant that a student whose credit score was below 60 should be rejected to get a loan. However, as a contrast experiment, loans were also approved to students with credit scores below 60.

### 5.2. Outcomes of the Lending Platform

Using the P2P lending platform, 258 loans were given out during ten months in the period of February 2016 to the end of December 2016. All the loans were repaid back finally. Among these debtors, the number of undergraduate students (which was 189) was much higher than the number of postgraduate students (which was 67), and there were only two doctoral students. Freshmen and sophomores accounted for more than half of total debtors, which turned out that freshmen and sophomores had less disposable money to meet their daily consumption or other expenses so that they had to turn to lending networks. In comparison, juniors and seniors may have more ways to earn pocket money, so relatively, they did not have large demand for loans. In addition, almost all debtors were science and engineering students (account for 94%). However, given the fact that this anonymous university is a prestigious university of science and engineering, we cannot conclude convincingly that science and engineering students have more demand for loans than students of art.

**Figure 2:**
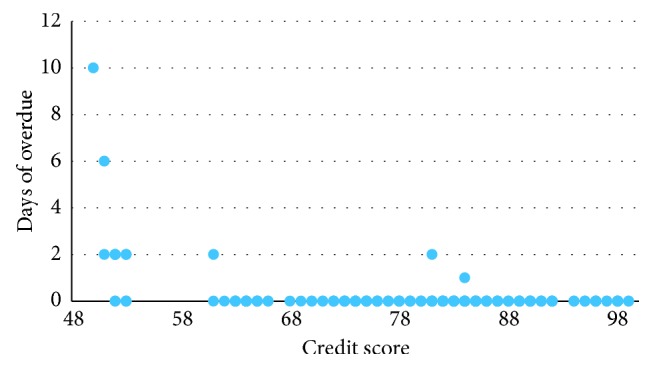
The scattering diagram of overdue days versus credit scores (some of the points overlap).

According to the rules of our credit model, students whose credit score were below 60 were “bad borrowers.” In this experiment, there were 13 predicted “bad borrowers.” Figure 2 illustrates the overdue days and credit scores of all users. There were 9 default cases.  Table 9 shows the number of debtors and overdue debtors in different levels of credit scores. It shows that the credit scores of the majorities were between 89 to 90, and among them, 2 debtors delayed for one day and two days separately. Another defaulter should be a “good borrower” who had a credit score of 61 delayed for 2 days. Comparatively, the predicted “bad borrowers” did not perform well, and there were 9 (out of 13) of them who were defaulters. Besides, the mean of overdue days was 3.67 days. There were 4 students who repaid two days after the deadline, while one (whose score was 51) delayed for 6 days and another (whose score was 50) delayed for 10 days. Obviously, the results were consistent with what our credit model predicted. If those 13 students were rejected, the default ratio would reduce from 3.5% to 1.2%, which is significant for risk controlling.

### 5.3. Experiment Limitations

The experiment is successfully completed based on a small-ranged P2P lending platform. However, there are still several limitations. First, this experiment is based on a condition that users do not have to pay any interest. Thus, it is not enough to infer what these students would behave if there was an interest rate. Secondly, the university in which the experiment is conducted is one of the top universities in China, and it may not be able to represent the general situation of all Chinese universities. Last, the scale of samples is too small. In order to examine the accuracy of our opinion, the range of this P2P lending platform should be developed in the future.

## 6. Conclusions

This study demonstrates that data mining meshed with machine learning algorithms, such as LR and GBDT, can perform well for the purpose of students' credit evaluation who have enrolled in a Chinese university. It was herein to creatively adopt the default of returning books as the index of financial default. Besides, two credit evaluation models were developed based on LR and GBDT algorithms separately. By comparing the achieved results, both models have shown excellent performance, while the GBDT-based model possessed higher capability on recognizing “bad borrower.” The result of P2P lending platform showed that employing a robust credit evaluation model is helpful to screen loan applications and can significantly prevent occurrence of default cases. While university students had a good credit in average, rejection of applicants with low credit scores was efficient to reduce the default ratio from 3.5% to 1.2%. To set up a robust credit evaluation system among different universities, several issues need to be solved. For instance, most of the Chinese universities do not have data collection or information storage systems. Data analysis procedure is based on large amounts of raw data; thus, it is of great importance to establish unified data collection systems. In addition, regarding the heterogeneity of different universities, the feature extraction process should be more holistic. Also, universities should enhance their relationships with social organizations not only to further develop the credit evaluation system but also to motivate their students to use credit services.

This study can be potentially generalized to other types of population. As for community finance, the problem of depicting the features and preferences of community population's expenditure behavior remained unsolved. Similar to university students, people in the same community have strong homogeneity. Only if the characters would be grabbed, product design and commercial promotion of local business can be more effective. Also, the proposed prediction methods can be applied to other university students' problems. Our team recently found a solution to foresee students' psychological problems by analyzing their reading information. It turns out the preference on book types has a strong association with one's psychology condition. Further research studies should be carried out on the issues associated with university students in the future.

## Figures and Tables

**Table 1:** The details of original features.

Variable	Meaning	Type	Count
Prob_Back	Each politic status's default ratio	Float	31586
Prob_Nation	Each nation's default ratio	Float	31586
Prob_age	Each age's default ratio	Float	31586
Prob_Sex	Each sex's default ratio	Float	31586
sum_subject	Sum amount of subjects	Int	31586
sum_grade	Sum of grades	Float	31586
avg_grade	Average of grades	Float	31586
excellent_times	Times of excellent in exams	Int	31586
fail_times	Times of failure in exams	Float	31586
excellent_rate	Ratio of excellent courses	Float	31586
fail_rate	Ratio of failed courses	Float	31586
Lib_in_times	Times of going into library	Int	31586
Lib_in_times_wk	Times of going into library on weekdays	Int	31586
Lib_in_times_wkd	Times of going into library on weekends	Int	31586
Lib_borrow_total_cnt	Times of borrowing	Int	31586
Lib_borrow_books	Sum acount of books borrowed	Int	31586
Lib_borrow_exceed_cnt	Count of returning books overdue	Int	31586
Lib_borrow_avg_exceed_cnt	Ratio of returning overdue	Float	31586
Lib_borrow_avg_exceed_time	Mean duration of overdue	Float	31586
Lib_renew_times	times of renewing book	Int	31586
Lib_renew_prob	Ratio of renewing book	Float	31586
Lib_forbidden_times	Times of forbidden to borrow	Int	31586
transaction_times	Times of transaction	Int	31586
avg_cost	Mean amount of transaction cost	Float	31586
sum_transaction_amount	Sum amount of transaction cost	Float	31586
sum_transaction_wkd_amount	Sum amount of transaction cost in weekends	Float	31586
sum_transaction_wkd_times	Times of transaction in weekends	Int	31586
sum_recharge	Sum amount of recharge	Float	31586
mean_recharge	Mean amount of recharge	Float	31586

**Table 2:** Confusing matrix of an example.

Observed	Predicted		
	Good/Bad		Sum
	Good	Bad	
Good/Bad			
Good	900	50	950
Bad	5	45	50
Sum	905	95	1000

**Table 3:** Confusing matrix of another example.

Observed	Predicted		
	Good/Bad		Sum
	Good	Bad	
Good/Bad			
Good	920	30	950
Bad	25	25	50
Sum	945	55	1000

**Table 4:** The maximized likelihood parameter estimation of 8 features after refinement.

Parameter	DOF	Estimation	Standard error	Wald	Pr
Intercept	1	−0.8254	0.217	14.4698	0.0001
*Prob_Back*	1	0.9089	0.2841	10.2376	0.0014
*Lib_renew_prob*	1	−0.2577	0.0785	10.7788	0.001
*fail_rate*	1	6.3658	3.3029	3.7148	0.0539
*Lib_borrow_avg_exceed_time*	1	0.0266	0.00668	15.8433	<0.0001
*Lib_borrow_total_cnt*	1	−0.0887	0.00877	102.4603	<0.0001
*sum_transaction_amount*	1	0.000101	0.00003	11.6087	0.0007
*avg_cost*	1	0.00317	0.000759	17.4164	<0.0001
*mean_recharge*	1	−0.00226	0.000795	8.0483	0.0046

**Table 5:** The prediction result of LR model on 20 test cases.

Test case	Total	Correct	TP	TN	FP	FN	ACC (%)	Sensitivity (%)	Specificity (%)
1	4000	3650	154	3496	304	46	91.25	77.00	92.00
2	4000	3650	154	3496	304	46	91.25	77.00	92.00
3	4000	3648	152	3496	304	48	91.20	76.00	92.00
4	4000	3650	154	3496	304	46	91.25	77.00	92.00
5	4000	3650	154	3496	304	46	91.25	77.00	92.00
6	4000	3686	152	3534	266	48	91.15	76.00	93.00
7	4000	3652	156	3496	304	44	91.30	78.00	92.00
8	4000	3648	152	3496	304	48	91.20	76.00	92.00
9	4000	3684	150	3534	266	50	92.10	75.00	93.00
10	4000	3650	154	3496	304	46	91.25	77.00	92.00
11	4000	3650	154	3496	304	46	91.25	77.00	92.00
12	4000	3686	152	3534	266	48	92.15	76.00	93.00
13	4000	3652	156	3496	304	44	91.30	78.00	92.00
14	4000	3648	152	3496	304	48	91.20	76.00	92.00
15	4000	3652	156	3496	304	44	91.30	78.00	92.00
16	4000	3652	156	3496	304	44	91.30	78.00	92.00
17	4000	3650	154	3496	304	46	91.25	77.00	92.00
18	4000	3648	152	3496	304	48	91.20	76.00	92.00
19	4000	3686	152	3534	266	48	92.15	76.00	93.00
20	4000	3686	152	3534	266	48	92.15	76.00	93.00
Mean	4000	3659	153.4	3505.5	294.5	46.6	91.47	76.70	92.25

**Table 6:** The prediction result of GBDT model on 20 test cases.

Test case	Total	Correct	TP	TN	FP	FN	ACC (%)	Sensitivity (%)	Specificity (%)
1	4000	3700	166	3534	266	34	92.50	83.00	93.00
2	4000	3700	166	3534	266	34	92.50	83.00	93.00
3	4000	3698	164	3534	266	36	92.45	82.00	93.00
4	4000	3698	164	3534	266	36	92.45	82.00	93.00
5	4000	3704	170	3534	266	30	92.60	85.00	93.00
6	4000	3702	168	3534	266	32	92.55	84.00	93.00
7	4000	3664	168	3496	304	32	91.60	84.00	92.00
8	4000	3664	168	3496	304	32	91.60	84.00	92.00
9	4000	3664	168	3496	304	32	91.60	84.00	92.00
10	4000	3700	166	3534	266	34	92.50	83.00	93.00
11	4000	3666	170	3496	304	30	91.65	85.00	92.00
12	4000	3702	168	3534	266	32	92.55	84.00	93.00
13	4000	3702	168	3534	266	32	92.55	84.00	93.00
14	4000	3700	166	3534	266	34	92.50	83.00	93.00
15	4000	3700	166	3534	266	34	92.50	83.00	93.00
16	4000	3700	166	3534	266	34	92.50	83.00	93.00
17	4000	3664	168	3496	304	32	91.60	84.00	92.00
18	4000	3702	168	3534	266	32	92.55	84.00	93.00
19	4000	3702	168	3534	266	32	92.55	84.00	93.00
20	4000	3702	168	3534	266	32	92.55	84.00	93.00
Mean	4000	3692	167	3525	276	33	92.29	83.50	92.74

**Table 7:** The continuous prediction performance of LR model.

Group	Total	BBD	GBD	Sensitivity (%)	Specificity (%)
1	200	154	46	77.00	1.21
2	200	13	187	83.50	6.13
3	200	10	190	88.50	11.13
4	200	5	195	91.00	16.26
5	200	7	193	94.50	21.34
6	200	3	197	96.00	26.53
7	200	5	195	98.50	31.66
8	200	1	199	99.00	36.89
9	200	1	199	99.50	42.13
10	200	1	199	100.00	47.37
11	200	0	200	100.00	52.63
12	200	0	200	100.00	57.89
13	200	0	200	100.00	63.16
14	200	0	200	100.00	68.42
15	200	0	200	100.00	73.68
16	200	0	200	100.00	78.95
17	200	0	200	100.00	84.21
18	200	0	200	100.00	89.47
19	200	0	200	100.00	94.74
20	200	0	200	100.00	100.00

*Note*. BBD is the number of bad borrowers that was detected from each group; GBD is the number of good borrowers that was detected from each group.

**Table 8:** The continuous prediction performance of GBDT model.

Group	Total	BBD	GBD	Sensitivity (%)	Specificity (%)
1	200	159	41	79.50	1.08
2	200	11	189	85.00	6.05
3	200	10	190	90.00	11.05
4	200	5	195	92.50	16.18
5	200	7	193	96.00	21.26
6	200	3	197	97.50	26.45
7	200	3	197	99.00	31.63
8	200	1	199	99.50	36.87
9	200	0	200	99.50	42.13
10	200	1	199	100.00	47.37
11	200	0	200	100.00	52.63
12	200	0	200	100.00	57.89
13	200	0	200	100.00	63.16
14	200	0	200	100.00	68.42
15	200	0	200	100.00	73.68
16	200	0	200	100.00	78.95
17	200	0	200	100.00	84.21
18	200	0	200	100.00	89.47
19	200	0	200	100.00	94.74
20	200	0	200	100.00	100.00

**Table 9:** The distribution of debtor and defaulter in scores with different ranges.

Score	*N*_l_	*N*_d_	${\bar{T}}_{\text{mean}}$
>90	37	0	0
(80, 90]	126	2	1.5
(70, 80]	53	0	0
(60, 70]	29	1	1.5
≤60	13	6	3.67
Sum	258	9	3

*Note.N*_l_ = the number of loaners; *N*_d_ = the number of defaulters; ${\bar{T}}_{\text{mean}}$ = sum (overdue days)/number of defaulters.

## Data Availability

The data used to support the findings of this study are restricted by the anonymous university's ethics review board in order to protect students' privacy. Data are available for researchers who meet the criteria for access to confidential data.
