# Corrigendum to “A Trial of Student Self-Sponsored Peer-to-Peer Lending Based on Credit Evaluation Using Big Data Analysis”

**DOI:** 10.1155/2019/9095675

**Published:** 2019-09-23

**Authors:** Yujiao Hou, Xiaofeng Ma, Guang Mei, Ning Wang, Weisheng Xu

**Affiliations:** College of Electronics and Information Engineering, Tongji University, Shanghai, China

In the article titled “A Trial of Student Self-Sponsored Peer-to-Peer Lending Based on Credit Evaluation Using Big Data Analysis” [1], the e-mail address of the corresponding author was given incorrectly as “wiesenxu@outlook.com.” It should be corrected to “xuweisheng@tongji.edu.cn.” This correction has been applied in place.
